# Orally Administered Rhamnan Sulfate from *Monostroma nitidum* Significantly Inhibits Melanoma Metastasis in Lungs and Aorta of Mice Implanted with B16 Cells

**DOI:** 10.3390/md24040126

**Published:** 2026-03-29

**Authors:** Keiichi Hiramoto, Masashi Imai, Masahiro Terasawa, Koji Suzuki

**Affiliations:** 1 Faculty of Pharmaceutical Sciences, Suzuka University of Medical Science, Minamitamagaki-cho 3500-3, Suzuka 513-8670, Mie, Japan; hiramoto@suzuka-u.ac.jp (K.H.); dp23001@st.suzuka-u.ac.jp (M.I.); terasawa@konanchemical.co.jp (M.T.); 2 Konan Chemical Manufacturing Co., Ltd., Kitagomizuka, Kusu-cho, Yokkaichi 510-0103, Mie, Japan

**Keywords:** rhamnan sulfate, *Monostroma nitidum*, B16 melanoma cells, metastasis, inflammation, EMT, PAR1, C57BL/6J mice

## Abstract

Tumor metastasis is closely associated with coagulation and inflammation, particularly via thrombin–PAR1 signaling. However, the potential of natural polysaccharides such as rhamnan sulfate (RS) to modulate these pathways and suppress metastasis remains unclear. We aimed to investigate the effects of orally administered RS derived from *Monostroma nitidum* on melanoma metastasis and its underlying mechanisms. Male C57BL/6J mice were orally administered water or RS daily. On day 8, saline or B16 melanoma cells were injected intravenously. Mice were treated for 21 days and divided into four groups (control, RS-only, M + W, and M + RS; n = 5/group). Metastasis and related molecular factors were analyzed in plasma, lung, and aortic tissues. Significant lung and aortic metastases were observed in the M + W group but were markedly suppressed in the M + RS group. RS reduced the expression of inflammatory factors (e.g., IL-6, PAR1), proteases, leukocyte activation markers, complement factors, angiogenic factors, and EMT-related factors. Conversely, thrombin, thrombomodulin, plasmin, TAFIa, and tight junction proteins were increased in RS-treated mice. RS suppresses melanoma metastasis by modulating thrombin–PAR1-mediated inflammation and associated pathways. These findings suggest RS as a potential therapeutic agent, although further mechanistic and clinical studies are required.

## 1. Introduction

Seaweeds are rich in essential nutrients—including iodine, iron, and various vitamins—and are a natural source of antioxidants, soluble and insoluble dietary fibers, and polysaccharides [1]. Previous studies have shown that seaweeds are functional foods with health-promoting properties. In particular, polysaccharides extracted from *Monostroma (M.) nitidum*, a green alga that grows wild or is cultivated in coastal areas of East Asia, including Japan, have been reported to improve various diseases, including those of the cardiovascular and metabolic systems.

Rhamnan sulfate (RS), a soluble fiber found in the intercellular matrix of *M. nitidum*, is primarily composed of linear rhamnose chains with side chains and contains 25–30% sulfate groups [2,3]. We previously demonstrated that RS enhances thrombin inhibition in the presence of antithrombin and exhibits anti-inflammatory activity by suppressing thrombin- and tumor necrosis factor (TNF)-α-induced tissue factor (TF) expression in vascular endothelial cells in vitro [4]. Furthermore, RS has been shown to exhibit anti-inflammatory effects on vascular endothelial cells in lipopolysaccharide (LPS)-treated mice [5], to exert anti-atherosclerotic effects by improving lipid metabolism and suppressing vascular inflammation [6], and to improve cognitive impairment in mice by suppressing hyperglycemia-induced hippocampal inflammation and microglial activation [7]. Collectively, these findings suggest that RS possesses anticoagulant and anti-inflammatory properties relevant to disease prevention.

Tumor metastasis is a multistep process that requires dynamic interactions between tumor cells and target tissues in the host. Highly metastatic melanoma cells express elevated levels of TF, the key initiator of the blood coagulation cascade, thereby promoting tumor metastasis through both coagulation-dependent and -independent mechanisms [8,9]. Activated blood coagulation factors such as thrombin and factor Xa play central roles in tumor growth and metastasis. Thrombin activates protease-activated receptor (PAR)1 on tumor and host tissue cells, and PAR1 signaling promotes tumor cell proliferation and angiogenesis [10], enhances tumor cell adhesion by increasing integrin expression [11], promotes cell invasion [12], and induces epithelial–mesenchymal transition (EMT) to facilitate metastasis [13]. Factor Xa, either alone or in complex with factor VIIa, activates PAR2 on tumor and host tissue cells, triggering pathways that enhance tumor cell proliferation, invasion, angiogenesis, and metastasis [14].

In this study, we investigated the effect of orally administered RS on the metastasis of highly metastatic B16 melanoma cells [15] implanted in male C57BL/6J mice. Metastatic progression to the lungs (which contain a dense microvasculature) and the aorta (a major blood vessel) was assessed. Furthermore, the mechanism of action of RS was analyzed by measuring metastasis-associated factors using immunohistochemistry and enzyme-linked immunosorbent assay (ELISA).

## 2. Results

### 2.1. Effect of RS Ingestion on Body Weight and Lung Metastasis in B16 Cell-Injected Mice

**Figure 1:**
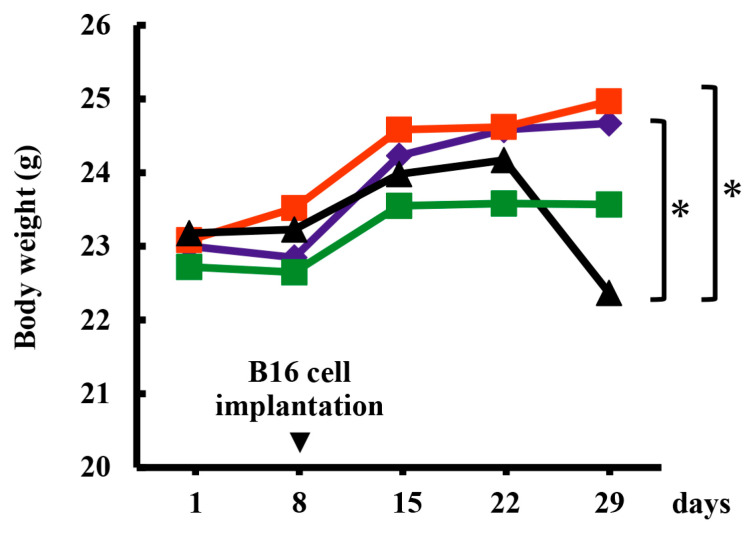
Effect of RS ingestion on the body weight of mice implanted with B16 cells. Body weight was measured weekly, and the average body weight of mice in each group is shown: control group (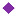), RS group (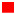), M + W group (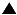), and M + RS group (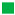). * *p* < 0.05.

Figure 1 shows the changes in the average body weight of mice in each group [water-only (W) group: control, RS-only (RS)-treated group, B16 melanoma cell (M) implantation followed by water (W) administration (M + W) group, and B16 melanoma cell implantation followed by RS administration (M + RS) group]. Comparing the body weights of the mice in each group on day 29, the weight of the M + W group was significantly lower than that of the control and RS groups (*p* < 0.05).

**Figure 2:**
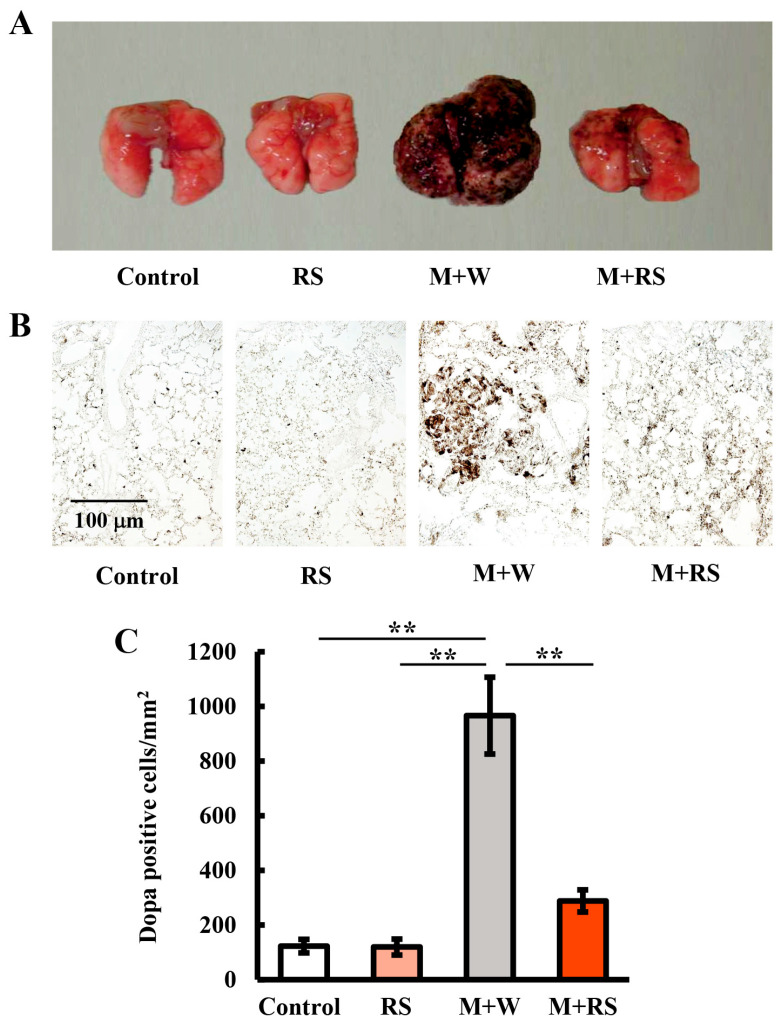
(**A**) Photographs of lung tissues from mice in each group, excised on day 29. Significant melanoma cell metastasis was observed in the lungs of mice in the M + W group, whereas metastasis was markedly suppressed in the lungs of mice in the M + RS group. (**B**) Distribution of DOPA-positive cells in the lung tissue from each group. (**C**) Images from each group shown in Figure 2B were analyzed, and the number of DOPA-positive cells per fixed area (mm^2^) was quantified. Statistical differences between groups are indicated as ** *p* < 0.01.

In mice in the M + W group, B16 cell metastasis was most clearly observed in the lungs; however, metastases were also observed in the liver, small intestine, large intestine, aorta, and mesenteric lymph nodes. Figure 2A shows photographs of the lungs of mice from each group excised on day 29. Significant B16 cell metastasis was observed in the lungs of mice in the M + W group, whereas metastasis was markedly suppressed in the lungs of mice in the M + RS group.

Figure 2B shows the distribution of 3,4-dihydroxyphenylalanine (DOPA)-positive cells in lung tissues. The number of DOPA-positive cells per fixed area (mm^2^), obtained by analyzing five or more images of lung tissue from each group, is shown in Figure 2C. The number of DOPA-positive cells in the M + W group was significantly increased (approximately 10-fold) compared to that in the control and RS groups, whereas it was significantly decreased (to approximately one-quarter) in the M + RS group compared to the M + W group.

### 2.2. Effect of RS Ingestion on Plasma and Lung Factors Related to Tumor Invasion and Metastasis in Mice Implanted with B16 Melanoma Cells

Tumor-bearing mice have been reported to exhibit elevated levels of inflammation, coagulation, fibrinolysis, matrix degradation, and tumor growth factors, all of which are involved in tumor invasion and metastasis. We measured several factors associated with tumor invasion and metastasis in the plasma and lung tissues of mice implanted with B16 cells using ELISA, and analyzed the effects of RS ingestion on these factors.

As shown in Table 1, plasma interleukin-6 (IL-6), urokinase-type plasminogen activator (uPA), matrix metalloprotease (MMP)-2 and MMP-9, as well as lung tissue cell membrane PAR1 and PAR2, and the tumor growth factor transforming growth factor (TGF)-β1 significantly increased in the M + W group compared to the control and RS groups (*p* < 0.01). However, IL-6, uPA, MMP-2, MMP-9, and PAR1 were significantly decreased (*p* < 0.05 or *p* < 0.01) in the M + RS group compared to the M + W group, whereas no significant decrease was observed in PAR2 and TGFβ1. The different effects of RS ingestion on the expression of PAR1 and PAR2, cell membrane receptors for coagulation factors, indicate that RS selectively modulates the thrombin–PAR1 pathway rather than the factor Xa–PAR2 pathway.

These results suggest that IL-6, uPA, MMP-2, MMP-9, and PAR1, which were reduced by RS ingestion, may be involved in the suppression of RS-dependent melanoma cell metastasis, whereas PAR2 and TGFβ1, which were not reduced, may not be involved. In particular, suppression of thrombin-mediated PAR1 activation in tissue cells may play an important role in the suppression of B16 cell invasion and metastasis by RS.

### 2.3. Effect of RS Ingestion on the Inflammation, Coagulation, and Fibrinolysis-Related Factors in Lungs and Aorta of Mice Implanted with B16 Melanoma Cells

Table 2 shows the results of the measurement of inflammatory-, coagulation-, and fibrinolysis-related factors in the lung tissues of mice in each group using ELISA or immunohistochemistry. The levels of TNFα and TF measured by ELISA were significantly increased (*p* < 0.01) in the lungs of mice in the M + W group compared to the control and RS groups, whereas these factors were significantly decreased (*p* < 0.01) in the M + RS group compared to the M + W group.

**Figure 3:**
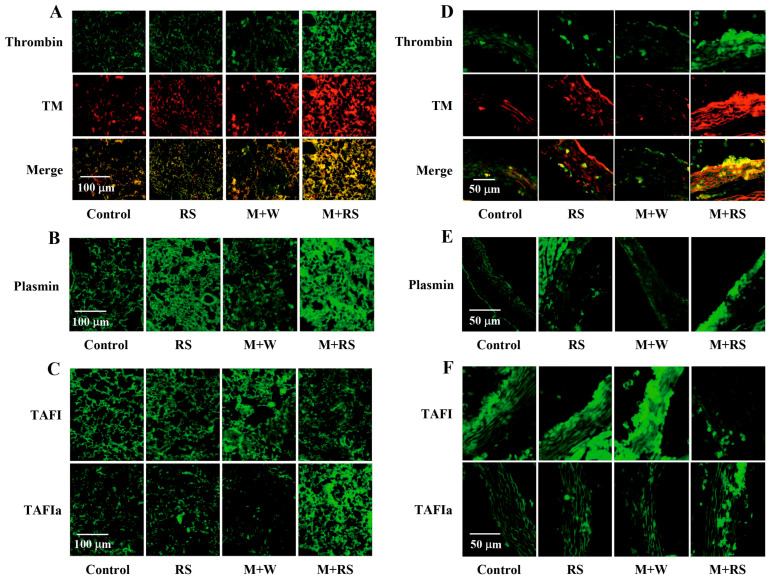
(**A**) Fluorescent immunohistochemical analysis of thrombin and thrombomodulin (TM) expression in the lung tissues of mice from each group. The bottom row shows merged images of both factors. (**B**) Fluorescent immunohistochemical analysis of plasmin expression in the lung tissues of mice from each group. (**C**) Fluorescent immunohistochemical analysis of TAFI and TAFIa expression in the lung tissues of mice from each group. (**D**) Fluorescent immunohistochemical analysis of thrombin and TM expression in the aorta tissues of mice from each group. The bottom row shows merged images of both factors. (**E**) Fluorescent immunohistochemical analysis of plasmin expression in the aorta tissues of mice from each group. (**F**) Fluorescent immunohistochemical analysis of TAFI and TAFIa expression in the aortic tissues of mice from each group.

Figure 3A shows immunohistochemical data for the distribution of thrombin and its functional regulator, thrombomodulin (TM), in the lungs of mice in each group, and the measured fluorescence intensity values are presented in Table 2. Thrombin and TM levels were unchanged in the M + W group compared to the control and RS groups but were significantly increased in the M + RS group (*p* < 0.01). Furthermore, merge analysis revealed that both factors colocalized, suggesting that these two factors, known to have high mutual affinity, form a complex.

The immunohistochemical data and measurements of the distribution of the fibrinolytic factor plasmin (Figure 3B) and the fibrinolytic regulatory factors thrombin-activatable fibrinolysis inhibitor (TAFI) and activated TAFI (TAFIa) (Figure 3C) in the lungs of mice from each group are shown in Table 2. Plasmin levels were significantly higher in the RS group than in the control group (*p* < 0.05), remained unchanged in the M + W group, and were significantly increased in the M + RS group (*p* < 0.01). In contrast, TAFI was significantly elevated in the M + W group compared to the control group (*p* < 0.01), and TAFIa was significantly elevated in the M + RS group (*p* < 0.01).

Figure 3D shows immunohistochemical data for the distribution of thrombin and TM in the aortas of mice from each group. Similar to lung tissue, thrombin and TM levels in the aorta of the M + RS groups were significantly elevated, and merged analysis suggested that the two factors co-localized and formed a complex. The distribution of plasmin (Figure 3E), TAFI, and TAFIa (Figure 3F) in the aortas of mice in each group was similar to that in the lung tissue.

Table 2 shows the expression levels of TNFα and TF measured by ELISA in mouse aortic tissues from each group, as well as the fluorescence intensity of coagulation- and fibrinolysis-related factors determined by immunohistochemical staining. TNFα, TF, and TAFI in the M + W group were significantly increased (*p* < 0.01) compared to the control and RS groups, whereas they were significantly decreased (*p* < 0.01) in the M + RS group compared to the M + W group. Thrombin, TM, plasmin, and TAFIa levels were significantly higher (*p* < 0.01) in the M + RS group than in the other groups.

TAFIa is a carboxypeptidase whose precursor molecule, TAFI, is specifically activated by the thrombin–TM complex and inhibits fibrinolysis by cleaving lysine residues on fibrin molecules, which are essential for fibrin degradation by plasmin. Therefore, the increase in plasmin and TAFIa in the M + RS group suggests that both the fibrinolytic system and the fibrinolysis regulatory system are enhanced in the lung and aortic tissues of the M + RS group.

### 2.4. Effect of RS Ingestion on the Expression of Tumor-Associated Leukocyte Factors in the Lungs and Aorta of B16 Cell-Implanted Mice

Tumor-associated leukocytes, such as tumor-associated macrophages and neutrophils, are abundant in the organs of tumor-bearing animals [16]. These cells produce various pro-inflammatory and pro-coagulant factors, such as TF, IL-6, tumor growth factors, and factors that promote tumor invasion and metastasis. In this study, we analyzed the effects of RS ingestion on macrophage accumulation and neutrophil activation in tumor-bearing lung and aortic tissues.

**Figure 4:**
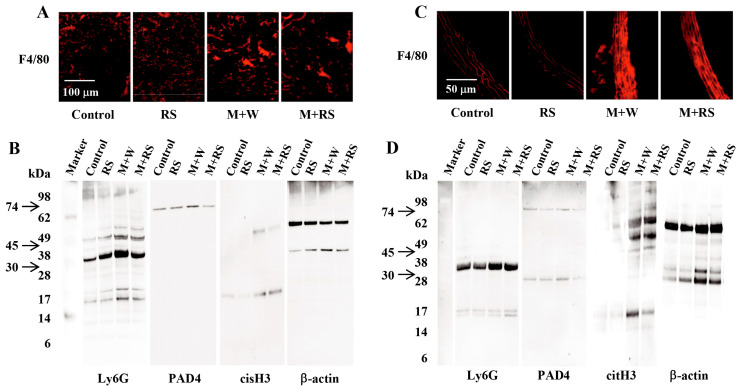
(**A**) Fluorescent immunohistochemical analysis of macrophage (F4/80) expression in the lung tissues of mice from each group. (**B**) Western blotting analysis of the expression levels of neutrophil activation markers (Ly6G, PAD4, and cisH3) and the control marker β-actin in the lung tissues of mice from each group. (**C**) Fluorescent immunohistochemical analysis of macrophage (F4/80) expression in the aorta tissues of mice from each group. (**D**) Western blot analysis of the expression levels of neutrophil activation markers (Ly6G, PAD4, and cisH3) and the control marker β-actin in the aorta tissues of mice from each group.

Figure 4A shows immunohistochemical data for F4/80 expression, which is specifically expressed on the surface of tumor-associated macrophages infiltrating the lung tumor tissue. As shown in Table 3, macrophage accumulation was significantly increased (*p* < 0.01) in the M + W and M + RS groups compared to the control and RS groups, whereas it was significantly suppressed (*p* < 0.05) in the M + RS group compared to the M + W group.

Figure 4B shows Western blot analysis of the expression of neutrophil activation markers lymphocyte antigen 6 complex locus G (Ly6G) (30 kDa), protein-arginine deiminase type-4 (PAD4) (74 kDa), and cytokine-inducible SH2-containing protein-3 (cisH3) (17 kDa), as well as the control marker β-actin (45 kDa), in the lungs of mice from each group. As shown in Table 3, the expression ratios of Ly6G, PAD4, and cisH3 to β-actin, calculated from the analytical data in Figure 4B, were significantly increased (*p* < 0.01) in the lungs of mice in the M + W group compared with the control and RS groups. In contrast, these markers were significantly decreased (*p* < 0.05 or *p* < 0.01) in the M + RS group compared with the M + W group.

The effect of RS ingestion on macrophage accumulation and neutrophil activation in the tumor-bearing aortic tissue was similar to that observed in the lungs. Figure 4C shows immunohistochemical data for macrophage expression in the aortic tissues of mice from each group. Figure 4D shows Western blot analysis of the expression of neutrophil activation markers Ly6G, PAD4, and cisH3, as well as the control marker β-actin, in the aorta of mice from each group.

As shown in Table 3, macrophage accumulation in aortic tissues was significantly increased (*p* < 0.01) in the M + W and M + RS groups compared to the control and RS groups, but was significantly lower (*p* < 0.05) in the M + RS group than in the M + W group. The expression ratios of Ly6G, PAD4, and cisH3 to β-actin were significantly increased (*p* < 0.01) in the M + W groups compared to the control and RS groups. Furthermore, except for the Ly6G/β-actin ratio, the PAD4/β-actin and cisH3/β-actin ratios were significantly decreased (*p* < 0.01) in the M + RS group compared to the M + W group. These results suggest that RS ingestion suppressed macrophage accumulation and leukocyte activation in the lung and aorta tissues of tumor-bearing mice.

### 2.5. Effect of RS Ingestion on the Expression of Complement System Factors in the Lungs and Aorta of B16 Cell-Implanted Mice

Tumor-associated macrophages surrounding tumor cells release complement component 1q (C1q), which binds to receptors on the tumor cell surface to form complexes with C1r and C1s. This complex activates C4 and C2 to form a C3 convertase, which converts C3 into C3a and C3b. C3a promotes tumor cell proliferation, metastasis, and angiogenesis, whereas C3b activates the complement pathway and promotes tumor cell proliferation. C3b also forms a complex with C4b and C2a to produce C5 convertase (C4bC2aC3b). C5 is converted into C5a and C5b; C5a promotes tumor cell proliferation and metastasis, whereas C5b inhibits these processes [17].

**Figure 5:**
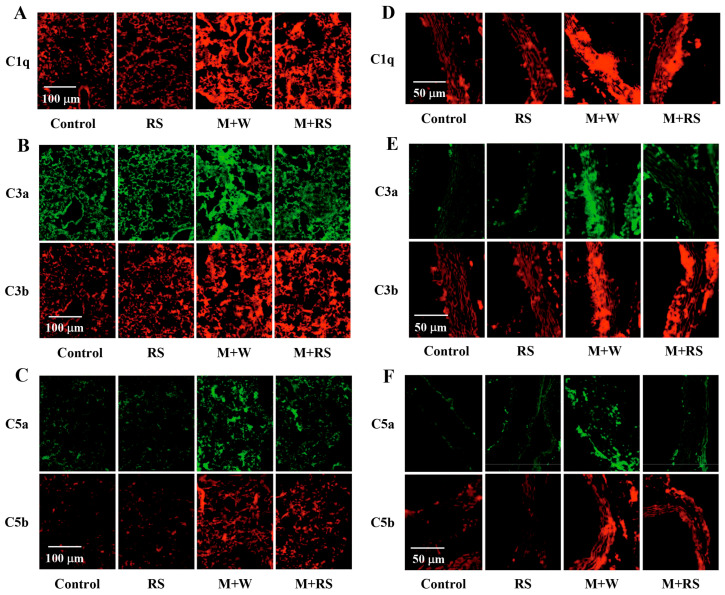
(**A**) Fluorescent immunohistochemical analysis of complement factor C1q expression in the lung tissues of mice from each group. (**B**) Fluorescent immunohistochemical analysis of C3a and C3b expression in the lung tissues of mice from each group. (**C**) Fluorescent immunohistochemical analysis of C5a and C5b expression in the lung tissues of mice from each group. (**D**) Fluorescent immunohistochemical analysis of C1q expression in the aorta tissues of mice from each group. (**E**) Fluorescent immunohistochemical analysis of C3a and C3b expression in the aorta tissues of mice from each group. (**F**) Fluorescent immunohistochemical analysis of C5a and C5b expression in the aortic tissues of mice from each group.

We analyzed the effect of RS ingestion on the expression of C1q, C3a, C3b, C5a, and C5b in the lung and aortic tissues of tumor-bearing mice using immunohistochemistry. Figure 5A–C show the changes in the expression of complement factors in the lung tissues. The fluorescence intensities obtained from the analyzed images are listed in Table 4. Lung C1q levels were significantly higher (*p* < 0.01) in the M + W and W + RS groups than in the control and RS groups. The C1q levels in the M + RS group were significantly lower (*p* < 0.05) than those in the M + W group.

The expression of C3a and C3b in lung tissues was significantly higher in the M + W group than in the control and RS groups. In the M + RS group, C3a expression was significantly lower (*p* < 0.01) than that in the M + W group, whereas C3b expression was not. The expression of C5a and C5b in lung tissues was significantly higher (*p* < 0.01) in the M + W group than in the control and RS groups; however, C5a expression in the M + RS group was significantly lower (*p* < 0.05) than that in the M + W group, whereas C5b expression was not.

Figure 5D–F show the changes in the expression of complement factors in aortic tissues. The fluorescence intensities of each factor obtained from the analyzed images are listed in Table 4. Similar to the findings in the lungs, aortic C1q levels were significantly higher (*p* < 0.01) in the M + W and W + RS groups and significantly lower (*p* < 0.05) in the M+RS group than in the M + W group. C3a and C3b levels were significantly higher (*p* < 0.01) in the M + W group, and C3a levels were significantly lower (*p* < 0.01) in the M + RS group than in the M + W group; however, C3b levels were not. Furthermore, the expression levels of C5a and C5b were significantly higher (*p* < 0.01) in the M + W group, whereas C5a expression in the M + RS group was significantly lower (*p* < 0.01) than that in the M + W group, but C5b expression was not.

### 2.6. Effect of RS Ingestion on the Expression of Angiogenesis-Related Factors in the Lungs and Aorta of B16 Cell-Implanted Mice

Many molecules, including MMPs, PARs, angiopoietin-2 (Ang-2), basic fibroblast growth factor (bFGF), and vascular endothelial growth factor, are involved in tumor angiogenesis, tissue invasion, and metastasis [18]. Furthermore, Roundabout homolog 4 (Robo4; an intercellular protein in vascular endothelial cells involved in tumor angiogenesis and inflammation), claudin 5 (an intercellular adhesion molecule that forms tight junctions and is involved in the regulation of angiogenesis), and E-cadherin (an intercellular adhesion molecule that forms tight junctions and is involved in cancer cell proliferation and metastasis) are also known to be involved in tumor invasion and metastasis.

**Figure 6:**
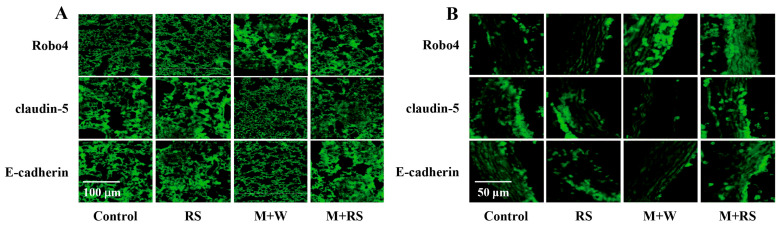
(**A**) Fluorescent immunohistochemical analysis of Robo4, claudin 5, and E-cadherin expression in the lung tissues of mice from each group. (**B**) Fluorescent immunohistochemical analysis of Robo4, claudin 5, and E-cadherin expression in the aortic tissues of mice from each group.

Since the levels of MMP-2, MMP-9, PAR1, PAR2, and TGFβ1 have already been presented in Table 1, we investigated the effects of RS ingestion on the levels of Ang-2, bFGF, Robo4, claudin 5, and E-cadherin in lung and aortic tissues from each group. Table 5 shows the levels of Ang-2 and bFGF measured by ELISA and the levels of Robo4, claudin 5, and E-cadherin quantified by immunohistochemistry. As shown in Figure 6A, the expression of Ang-2, bFGF, and Robo4 in lung tissue was significantly increased (*p* < 0.01) in the M + W group and significantly decreased (*p* < 0.05 or *p* < 0.01) in the M + RS group compared to the M + W group. Meanwhile, the expression levels of claudin 5 and E-cadherin were significantly lower (*p* < 0.01) in the M + W group than in the control and RS groups, and significantly higher (*p* < 0.01) in the M + RS group than in the M + W group.

The effects of RS ingestion on the expression of angiogenesis-related factors in aortic tissues were similar to those observed in the lungs. As shown in Figure 6B and Table 5, Ang-2, bFGF, and Robo4 were significantly increased (*p* < 0.01) in the M + W group and significantly decreased (*p* < 0.01) in the M + RS group compared to the M + W group. However, the expression levels of the tight junction proteins claudin 5 and E-cadherin were significantly decreased (*p* < 0.01) in the M + W group compared to the control and RS groups, and significantly increased (*p* < 0.01) in the M + RS group compared to the M + W group.

These results indicate that tumor angiogenesis-related factors were increased and intercellular tight junction proteins were decreased in the lung and aortic tissues of B16–cell implanted mice, whereas RS ingestion suppressed tumor angiogenesis by decreasing angiogenic factors and increasing tight junction proteins.

### 2.7. Effect of RS Ingestion on the Expression of EMT-Related Factors in the Lungs and Aorta of Mice Implanted with B16 Cells

EMT, a process by which epithelial cells acquire mesenchymal cell properties, plays an important role in cancer cell invasion into surrounding tissues and distant metastasis [19]. EMT-related factors include extracellular matrix-related proteins such as β-catenin, vimentin, and fibronectin, as well as transcription factors such as Snail-1 and Wnt. MMPs, whose expression is induced by EMT-related transcription factors, are activated by plasmin and other proteases and promote EMT by degrading tight junction proteins between epithelial cells.

Table 6 shows the expression levels of β-catenin, fibronectin, Snail-1, and vimentin in the lung tissues of mice in each group using ELISA. These EMT-related factors were significantly increased (*p* < 0.05 or *p* < 0.01) in the M + W group compared to the control and RS groups and were significantly decreased (*p* < 0.05 or *p* < 0.01) in the M + RS group compared to the M + W group.

Both Wnt3a and Wnt5a are ligands that activate the Wnt signaling pathway and mediate EMT, but their activation pathways and cellular responses differ [20,21]. Wnt3a is involved in β-catenin-mediated cell proliferation and differentiation, whereas Wnt family member 5A (Wnt5a) is involved in β-catenin-independent regulation of cell proliferation and differentiation.

**Figure 7:**
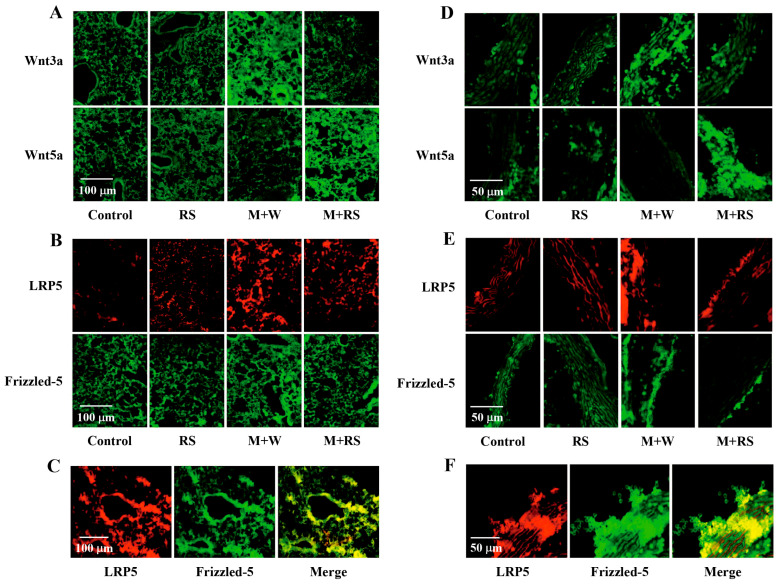
(**A**) Fluorescent immunohistochemical analysis of Wnt3a and Wnt5a expression in the lung tissues of mice from each group. (**B**) Fluorescent immunohistochemical analysis of LRP5 and Frizzled-5 expression in the lung tissues of mice from each group. (**C**) Fluorescent immunohistochemical analysis of LRP5 and Frizzled-5 expression in the lung tissues of mice in the M + W group, including merged images of both factors. (**D**) Fluorescent immunohistochemical analysis of Wnt3a and Wnt5a expression in the aortic tissues of mice from each group. (**E**) Fluorescent immunohistochemical analysis of LRP5 and Frizzled-5 in the aortic tissues of mice from each group. (**F**) Fluorescent immunohistochemical analysis of LRP5 and Frizzled-5 expression in the aortic tissues of mice in the M + W group, including merged images of both factors.

Figure 7A shows immunohistochemical data for wingless-type MMTV integration site family member 3A (Wnt3a) and Wnt5a expression in lung tissue from each group. As shown in Table 6, Wnt3a expression levels were significantly higher (*p* < 0.01) in the M + W group, whereas Wnt5a expression levels were significantly higher (*p* < 0.01) in the M + RS group. This suggests that B16 cell proliferation and metastasis in the M + W group were mediated by a β-catenin-dependent increase in Wnt3a, whereas the suppression of B16 cell proliferation and metastasis in the M + RS group was associated with a β-catenin-independent increase in Wnt5a.

Low-density lipoprotein receptor-related protein 5 (LRP5) functions as a co-receptor with Frizzled-5 receptors in the Wnt/β-catenin pathway and is involved in cancer cell proliferation, invasion, and metastasis. On the other hand, Frizzled- 5 is known to induce EMT by activating the signal transducer and activator of transcription and the mitogen-activated protein kinase/extracellular regulated kinase pathways in a β-catenin-independent manner upon binding of its ligand, Wnt.

Figure 7B shows immunohistochemical data for LRP5 and Frizzled-5 expression in lung tissue from each group. As shown in Table 6, the expression levels of both factors were significantly increased (*p* < 0.01) in the M + W group compared to the control and RS groups and significantly decreased (*p* < 0.01) in the M + RS group compared to the M + W group. Furthermore, as shown in Figure 7C, merged image analysis of both factors, whose expression levels were elevated in the M + W group, suggested that they were expressed in the same tissue. These results suggest that extracellular Wnt ligands bind to LRP5 and Frizzled-5 as coreceptors and activate the Wnt signaling pathway in lung tissue.

The effects of RS ingestion on the expression of EMT-related factors in the aortic tissue of tumor-bearing mice were similar to those observed in the lungs. Table 6 shows the expression levels of EMT-related factors measured by ELISA and immunohistochemistry in the aortic tissues of mice from each group.

The expression levels of β-catenin, fibronectin, Snail-1, and vimentin in the aorta were all significantly increased (*p* < 0.01) in the M + W group and significantly decreased (*p* < 0.05 or *p* < 0.01) in the M + RS group compared to the M + W group. Furthermore, as shown in Figure 7D, Wnt3a expression levels were significantly increased in the M + W group, whereas Wnt5a expression levels were significantly increased in the M + RS group.

As shown in Figure 7E, the expression levels of LRP5 and Frizzled-5 were significantly increased in the M + W group but significantly decreased in the M + RS group compared to the M + W group. Furthermore, as shown in Figure 7F, merged image analysis of LRP5 and Frizzled-5, whose expression levels were increased in the M + W group, suggested that they were expressed in the same tissue cells. These data indicate that extracellular Wnt ligands bind to LRP5 and Frizzled-5 as co-receptors and activate the Wnt signaling pathway in aortic tissue.

These results indicate that all EMT-promoting factors examined in this study (except for Wnt5a, which acts to suppress EMT) were increased in the lung and aortic tissues of tumor-bearing mice, and that these increases were suppressed by RS ingestion.

## 3. Discussion

Highly metastatic melanoma cells express large amounts of TF, an initiator of the blood coagulation cascade that significantly enhances the coagulation and inflammatory responses of the host [8]. Melanoma cell metastasis is promoted in a coagulation-dependent manner, and activated coagulation factors, thrombin and factor Xa, play important roles in tumor growth and metastasis [8,21]. The thrombin receptor PAR1 and factor Xa receptor PAR2 are involved in tumor cell growth and metastasis and are widely expressed on both tumor cells and host tissue cells [10,22]. Thrombin increases the expression of PAR1 [13], whereas factor Xa increases the expression of PAR2. These PAR1- and PAR2-mediated signaling pathways activate different transcription factors and genes, thereby promoting tumor proliferation, increasing the expression of adhesion molecules [11], promoting tumor cell adhesion and tissue invasion [12], inducing EMT in surrounding tissues and tumor angiogenesis, and ultimately promoting metastasis [14,23]. Furthermore, the fibrinolytic system, involving plasmin and its regulatory factors, and the complement system, which induces host inflammation and promotes cancer cell proliferation and metastasis, play important roles in tumor angiogenesis, cancer progression, and tumor cell destruction associated with cancer therapy [24].

We previously found that edoxaban, a factor Xa inhibitor among direct oral anticoagulants (such as dabigatran etexilate, rivaroxaban, and edoxaban), reduced PAR2 expression and suppressed coagulation and inflammatory responses via the PAR2 signaling pathway, thereby inhibiting tumor growth in mice implanted with non-metastatic colon cancer cells (Colon26 cells) [25]. This study showed that edoxaban induces apoptosis by enhancing the PAR1 pathway, which antagonizes the PAR2 pathway. Furthermore, we recently reported that edoxaban inhibits the metastasis of highly metastatic B16 melanoma cells by suppressing the PAR2 pathway and its downstream TGFβ pathway and inhibits EMT and angiogenesis in cells surrounding B16 cells [26]. This study also showed that the thrombin inhibitor dabigatran etexilate significantly suppressed B16 cell metastasis by inhibiting the PAR1 pathway, although the inhibitory effect was less than that of edoxaban.

In the present study, we investigated the effects of orally administered RS on B16 cell metastasis in mice implanted with B16 cells. The mean body weight of mice in the M + W group on day 29 was significantly lower than that of mice in the control and RS groups, whereas the M + RS group showed a tendency toward improvement, although the difference was not significant. Furthermore, the M + RS group significantly suppressed the increase in B16 cell (DOPA-positive cell) clusters in the lung tissue.

Factors related to inflammation, tumor invasion, and metastasis in plasma (IL-6, uPA, MMP-2, and MMP-9) and lung tissue (PAR1, PAR2, and TGFβ1) were all significantly increased in the M + W group. However, in the M + RS group, all plasma factors and PAR1 levels were significantly decreased, whereas PAR2 and TGFβ1 were not. PAR2 and TGFβ1 are known to be interdependently involved in tumor angiogenesis and metastasis [26,27,28], suggesting that RS ingestion does not affect the factor Xa-PAR2-dependent pathway or the PAR2-TGFβ1-dependet pathway. In contrast, RS ingestion may suppress the activation and expression of PAR1 via thrombin produced in the host, thereby suppressing B16 cell invasion and metastasis.

Regarding factors related to inflammation, coagulation, and fibrinolysis in both lung and aortic tissues, TNFα, TF, and the inactive form of TAFI were significantly increased in the M + W group and significantly decreased in the M+RS group compared to the M + W group. The expression levels of thrombin, TM, and plasmin in the M + W group remained unchanged, whereas these factors, along with TAFIa, were significantly increased in the M + RS group. Merged image analysis of thrombin and TM suggests that they may form a complex in the same tissue, contributing to TAFI activation, as the thrombin-TM complex is known to activate TAFI [29]. These results suggest that RS ingestion enhances both fibrinolysis activation (increased plasmin) and fibrinolysis regulation (increased TAFIa) in melanoma-bearing mice, although the underlying mechanisms remain unclear.

Tumor-associated leukocyte (macrophages and activated neutrophils)-related factors were significantly increased in the lung and aortic tissues of the M + W group, whereas most of these factors were significantly decreased in the M + RS group. Tumor-associated macrophages are known to release C1q, which activates the complement system. The complement system is closely linked to the coagulation and fibrinolysis systems, and C3b forms complexes with factor H and regulates the complement system via TM [30]. Furthermore, TAFIa, activated by the thrombin–TM complex or plasmin, proteolytically inactivates C3a, C5a, and bradykinin, thereby suppressing vascular inflammation [31].

All complement factors (C1q, C3a, C3b, C5a, and C5b) measured in this study were significantly increased in the lung and aorta tissues of the M + W group. In the M + RS group, C3b and C5b did not show significant changes compared to the M + W group, whereas C1q, C3a, and C5a were significantly decreased, with particularly marked reductions in C3a and C5a. The increased levels of plasmin, thrombin–TM complex, and TAFIa suggest that degradation of C3a and C5a by TAFIa may be involved. These results suggest that RS ingestion may inhibit melanoma cell proliferation and metastasis by promoting the degradation of C3a and C5a while maintaining C3b and C5b levels.

Next, we analyzed the effects of RS ingestion on angiogenesis-related factors derived from tumor cells and surrounding tissue cells and their involvement in B16 cell invasion and metastasis. The levels of Ang-2, bFGF, and Robo4, as well as TGFβ1, MMP-2, and MMP-9, which are involved in tumor-associated angiogenesis, vascular permeability, and tumor invasion, were significantly increased in the M + W group compared to the control group. In contrast, claudin 5 and E-cadherin, which are involved in maintaining tight junctions between epithelial cells, were significantly decreased in the M + W group but significantly increased in the M + RS group. These findings suggest that RS ingestion suppresses B16 cell invasion into surrounding tissues and tumor-associated angiogenesis by increasing the expression of intercellular tight junction molecules such as claudin 5 and E-cadherin.

Finally, we analyzed the effects of RS ingestion on the expression of EMT-related factors, which are closely associated with tumor invasion and metastasis, in the tissues of B16 cell-implanted mice. The results showed that the expression of β-catenin, vimentin, Snail-1, and Wnt3a, which are involved in promoting EMT, was significantly increased in the lung and aortic tissue of the M + W group. This increase in expression was consistent with the increase in the expression of uPA, which promotes tumor cell migration, invasion, and proliferation by converting plasminogen to plasmin, activating MMPs, and degrading the extracellular matrix. In contrast, Wnt5a, which is involved in suppressing EMT, was significantly decreased. In the M + RS group, all factors except Wnt5a were significantly decreased, indicating that RS ingestion affects the expression of EMT-related factors associated with melanoma cell metastasis. Furthermore, the fact that RS ingestion significantly decreased Wnt3a expression and significantly increased Wnt5a expression suggests that the thrombin-PAR1 signaling pathway may be involved in the regulation of the Wnt signaling pathway. In addition, the expression levels of LRP5 and Frizzled-5, which are involved in the Wnt signaling pathway, were increased in the M + W group and decreased in the M + RS group. Image analysis integrating the expression of these two factors suggested that both factors are co-expressed within the same cell. These results suggest that RS ingestion reduces the binding of extracellular Wnt ligands to LRP5 and Frizzled-5 coreceptors in vascular endothelial cells of tumor-carrying mice, thereby suppressing the Wnt/β-catenin signaling pathway that promotes EMT.

**Figure 8:**
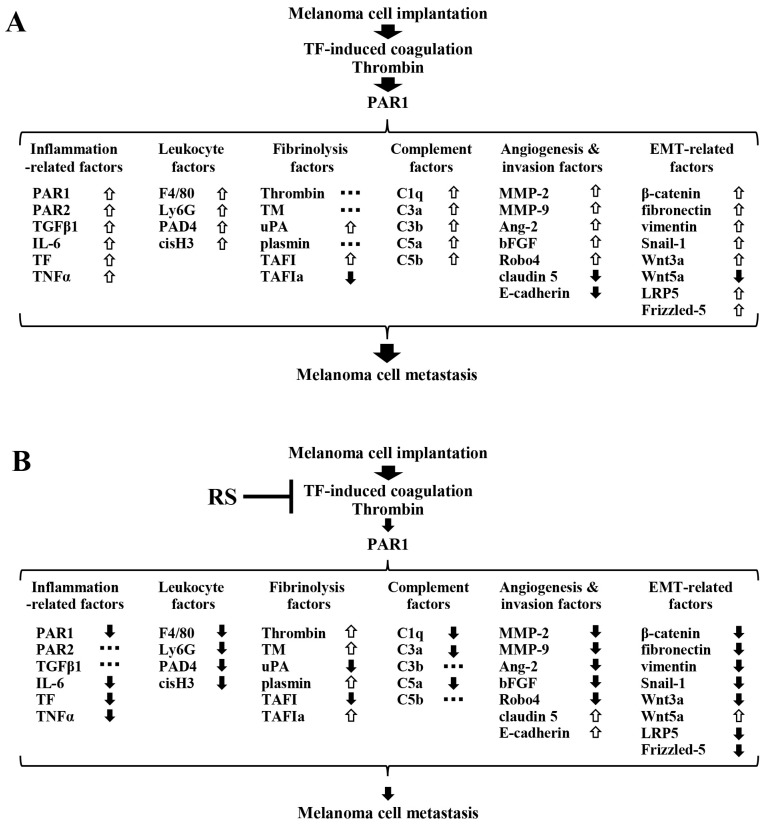
(**A**) Changes in the expression of melanoma cell metastasis-related factors in the M + W group mice. The expression levels of melanoma cell metastasis-related factors in the blood and tissues of M + W group mice implanted with B16 cells were compared to those in the control group. Factors that significantly increased are indicated by upward arrows, factors that remained unchanged are indicated by horizontal dotted lines, and factors that significantly decreased are indicated by downward arrows. The combined effects of changes in the expression of each factor are thought to promote melanoma cell metastasis. (**B**) Changes in the expression of melanoma cell metastasis-related factors in the M + RS group mice. The expression levels of melanoma cell metastasis-related factors in the blood and tissues of the M + RS group mice implanted with B16 cells were compared to those in the M + W group. Factors that significantly increased are indicated by upward arrows, factors that remained unchanged are indicated by horizontal dotted lines, and factors that significantly decreased are indicated by downward arrows. The combined effects of changes in the expression of each factor are thought to suppress melanoma cell metastasis. Abbreviations: PAR, protease-activated receptor; TGFβ1, transforming growth factor β1; IL-6, interleukin 6; TF, tissue factor; TNFα, tumor necrosis factor α; F4/80, macrophage marker F4/80; Ly6G, lymphocyte antigen 6 complex locus G6D; PAD4, peptidylarginine deiminase 4; cisH3, citrullinated Histone H3; TM, thrombomodulin; uPA, urokinase-type plasminogen activator; TAFI, thrombin-activatable fibrinolysis inhibitor; TAFIa, activated TAFI; MMP, matrix metalloproteinase; Ang-2, angiopoietin-2; bFGF, basic fibroblast growth factor; Robo4, Roundabout homolog 4; E-cadherin, epithelial cadherin; Snail-1, small family zinc finger 1; Wnt, wingless and int-1; Wnt3a, Wnt family member 3a; LRP, low-density lipoprotein receptor-related protein; Frizzled, Frizzled class receptor.

Based on these considerations, Figure 8A shows that, in mice in the M + W group, TF derived from implanted B16 cells induced blood coagulation, and the generated thrombin activated PAR1 in vasculature cells, causing changes in the expression of factors related to inflammation, leukocyte activation, fibrinolysis, complement, tumor angiogenesis and tissue invasion, and EMT. These changes collectively promote B16 cell metastasis. Upward arrows indicate increased expression, downward arrows indicate decreased expression, and horizontal arrows indicate no change in expression compared to the control group. Among these factors, only TAFIa, claudin 5, E-cadherin, and Wnt5a were significantly decreased compared to the control group, whereas all other factors, except thrombin, TM, and plasmin, were significantly increased compared to the control group.

Figure 8B shows factors whose expression levels were significantly altered in the M + RS group compared to the M + W group. These changes in factors are thought to be involved in the suppression of metastasis of implanted B16 cells by RS ingestion. Upward arrows indicate increased expression, downward arrows indicate decreased expression, and horizontal arrows indicate no change in expression. All inflammatory and leukocyte activation-related factors, except for PAR2 and TGFβ1, were significantly decreased. Among fibrinolysis- and complement-related factors, uPA, TAFI, C1q, C3a, and C5a were significantly decreased, whereas C3b and C5b remained unchanged. Thrombin, TM, plasmin, and TAFIa were significantly increased.

Regarding tumor angiogenesis- and tissue invasion-related factors, the tight junction molecules claudin 5 and E-cadherin were increased, whereas all other pro-angiogenic factors were significantly decreased. Furthermore, among EMT-related factors, Wnt5a, which inhibits EMT, was increased, whereas all other EMT-promoting factors were decreased. Changes in the expression of these factors induced by RS ingestion collectively suppressed the metastasis of implanted B16 cells.

The effects of RS are dose-dependent. Orally administered RS (0.25–2.5 mg RS /mouse) dose-dependently suppressed the increased vascular permeability observed in LPS-treated mice, restoring it to control levels. It also reduced various inflammatory markers, such as IL-6 and TF, demonstrating a dose-dependent anti-inflammatory effect of RS [5]. Furthermore, orally administered RS (0.25–7.5 mg RS/mouse) dose-dependently suppressed the increase in blood TNFα levels in hyperglycemic diabetic mice, whereas excessive RS administration attenuated this effect [7]. These results suggest that the bio-protective effect of RS depends on the appropriate amount of RS ingested.

The mechanisms by which orally administered RS suppresses tumor cell-derived TF-induced blood coagulation and tissue inflammation in mice implanted with B16 cells are unclear, but previous studies by our group and others have suggested several possibilities.

First, RS inhibits thrombin through an antithrombin-dependent mechanism [4]. In vitro experiments using purified coagulation factors have shown that RS potently inhibits thrombin in an antithrombin-dependent manner, like unfractionated heparin, while inhibiting factor Xa very weakly. Furthermore, experiments using cultured human umbilical vein endothelial cells have shown that RS dose-dependently inhibits the increased expression of TF and the platelet aggregation factor von Willebrand factor in unstimulated endothelial cells and in endothelial cells stimulated with thrombin, TNFα, or LPS.

Second, orally administered RS in mice potently suppresses endothelial cell inflammation [5]. RS suppressed intraperitoneally administered LPS-induced vascular hyperpermeability and neutrophil infiltration into organs, including the lungs and liver, and significantly reduced plasma levels of the inflammatory molecular markers, IL-6 and TF. Furthermore, RS maintained the expression level of syndecan-4 in endothelial cells and significantly inhibited the loss of the glycocalyx layer, thereby suppressing inflammatory damage to endothelial cells.

Third, RS inhibits hyaluronidase in a concentration-dependent manner [32]. Hyaluronidase degrades and strips hyaluronic acid, the main component of the glycocalyx layer of vascular endothelial cells, increasing vascular permeability and inducing endothelial dysfunction. Therefore, RS may play a role in protecting the vascular endothelium by inhibiting hyaluronidase.

Another possibility is that RS ingestion in humans modulates the composition of the gut microbiota, improves intestinal function, and suppresses systemic inflammation [33]. Indeed, administration of RS to mice has been shown to induce a healthy gut microbiota, which may be beneficial for health [34].

A limitation of this study was the inability to directly measure the blood concentrations of RS and its metabolites after oral administration. Therefore, it was not possible to determine the systemic distribution of RS or the amount of active RS present in the lungs and aorta. However, previous studies have confirmed that orally administered fluorescein isothiocyanate-labeled RS co-localizes with M cells and is taken up by Peyer’s patches [35], suggesting the possibility of intestinal absorption of RS. This possibility is supported by studies on fucoidan, another high molecular weight sulfated polysaccharide. Orally administered fucoidan has been detected in serum and urine and has been reported to be taken up by the intestines and liver tissue despite its low intestinal permeability [36,37]. Therefore, although direct pharmacokinetic data of RS are lacking, it is quite possible that some orally administered RS is absorbed into the body, transported to immune-related tissues, and thereby contributes to the systemic suppression of melanoma cell metastasis.

In conclusion, the results of this study suggest that orally administered RS inhibits thrombin generated during blood coagulation induced by implanted B16 cells via antithrombin, thereby suppressing the activation and expression of PAR1 in tissue cells and inhibiting vascular endothelial inflammation and complement system activation. RS also increases TAFIa production via plasmin and the thrombin–TM, thereby regulating tissue fibrinolysis. These effects of RS may collectively inhibit EMT and tumor angiogenesis, thereby suppressing melanoma cell invasion and metastasis.

Although this study was conducted using mice, the results suggest that oral administration of RS may suppress tumor growth and metastasis in patients with cancers such as melanoma. Further basic research and subsequent clinical studies are needed to understand the inhibitory effects of RS and other seaweed components on cancer cell growth and metastasis, their mechanisms of action, and their impact on human patients.

## 4. Materials and Methods

### 4.1. Animals and Melanoma Cells

Allogeneic transplantation studies were performed using specific pathogen-free (SPF) male 8-week-old C57BL/6J mice (SLC, Hamamatsu, Japan). Mice were housed in individual cages in an air-conditioned, SPF-controlled room at 23 ± 1 °C with a 12 h light/dark cycle (lights on at 08:00). Animals had ad libitum access to food and water.

A metastatic mouse melanoma cell line, B16 cells, established from a C57BL/6 mouse tumor [15] and obtained from the Japanese Collection of Research Bioresources Cell Bank (Osaka, Japan), was used at passages 5 to 15. The cells were cultured in Eagle’s minimum essential medium (Sigma-Aldrich, Darmstadt, Germany) supplemented with 10% serum and l-glutamine. The cells were tested periodically to ensure that they were free of Mycoplasma, mouse viruses, or tumorigenic contamination. Subconfluent monolayers were harvested after treatment with 1 mM 0.25% trypsin and 0.02% ethylenediaminetetraacetic acid (Sigma-Aldrich). The trypsinized cells were washed and resuspended in phosphate-buffered saline (PBS; Ca^2+^, Mg^2+^-free; Sigma-Aldrich Chemical, St. Louis, MO, USA).

### 4.2. RS Sample

RS is composed of α-1,3-linked L-rhamnose residues, some of which are sulfated mainly at the O-2 position, with trace amounts of 1,2-rhamnose and branched rhamnose residues [3]. In this study, we used an RS sample (purity 94%, molecular weight ranging from tens of thousands to hundreds of thousands, with an average molecular weight of approximately 150 kDa, and approximately 32% sulfate groups) purified from hot-water extracts of *M. nitidum* by Konan Chemical Manufacturing Co., Ltd. (Yokkaichi, Mie, Japan) using a previously published method [6].

### 4.3. Experiment on the Effect of Orally Administered RS on B16 Cell Metastasis in Mice and Sample Collection

**Figure 9:**
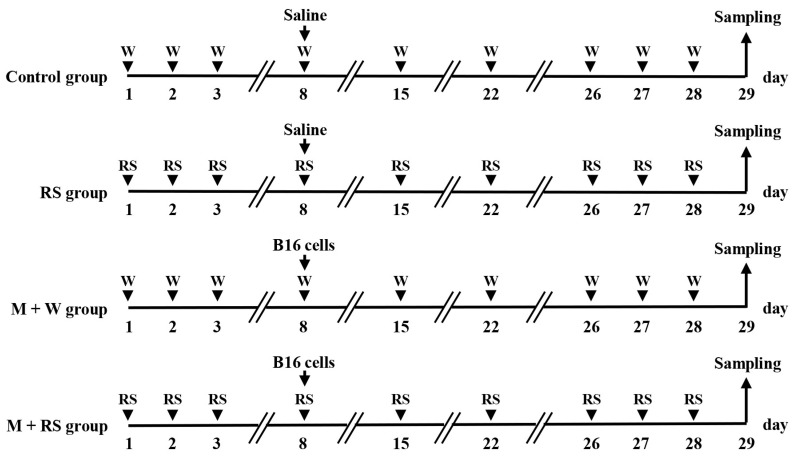
Experimental design to evaluate the effect of rhamnan sulfate (RS) on the metastasis of B16 cells implanted into C57BL/6J mice. Eight-week-old male C57BL/6J mice were orally administered with water or RS (6 mg/mouse) daily. On day 8, saline or B16 cells (M) (1 × 10^6^ cells) were injected into the tail vein. Subsequently, mice were orally administered water or RS daily for 21 days. Four groups were established: water-only (W) control, RS-only, M + W, and M + RS groups (*n* = 5/group). On day 29, blood was collected, and the lungs and aorta were excised to analyze the effects of RS administration on factors related to melanoma cell metastasis in plasma and tissues, as well as the underlying mechanisms.

Regarding the oral dosage of RS in mice, previous studies have shown that a dose of approximately 230 mg/kg body weight per day (6 mg per mouse) sufficiently improves inflammatory disorders without causing side effects such as bleeding [5,6,7], and this dosage was used in this study. An outline of the experimental method is shown in Figure 9.

Eight-week-old male C57BL/6J mice were orally administered water or RS (6 mg/100 μL/day) daily. On day 8, saline (100 μL) or B16 melanoma cells (M) (1 × 10^6^ cells/100 μL) were injected into the tail vein, and water or RS was then administered daily by feeding needle for 21 days to create four groups; water-only (W) group (control), RS-only group, M + W group, and M + RS group, *n* = 5/group).

Body weight was measured on days 1, 8, 15, 22, and 29. On day 29, the mice were anesthetized, and blood and organs were collected. Blood collected from the aorta via abdominal incision was mixed with 1/10 volume of 100 IU/mL heparin sodium and centrifuged at 3000× *g* at 4 °C for 10 min using a refrigerated centrifuge. The heparinized plasma was stored at −80 °C for later use. After blood collection, the lungs and aorta were harvested, photographed, and frozen at −80 °C for later use. Organ tissue samples were fixed in PBS containing 4% paraformaldehyde (Fujifilm Wako Pure Chemicals, Osaka, Japan).

### 4.4. Identification of Melanoma Cells in Organ Tissues and Immunohistochemical Analysis of Tumor-Associated Factors

Fixed tissue specimens were embedded in frozen Tissue–Tek OCT Compound (Sakura Finetek, Tokyo, Japan) and sliced into 5 µm thick sections. To identify melanoma cells in the tissue, DOPA-positive cells, which indicate melanocyte tyrosinase activity, were examined. DOPA-positive melanocytes were stained as previously described [26]. The tissue was washed with PBS and incubated in PBS containing 0.1% L-DOPA at 37 °C (Sigma-Aldrich Chemical, St. Louis, MO, USA). The specimens were washed with PBS for microscopic examination.

To visualize tumor-associated factors using immunohistochemical analysis, tissue specimens were incubated with factor-specific antibodies: rabbit polyclonal anti-thrombin (1:100; GTX610270, GeneTex, Irvine, CA, USA), rabbit polyclonal anti-plasmin (1:100; ABV11485, ABGENT, San Diego, CA, USA), rabbit polyclonal ant-TAFI (1:100; 55201-1-AP, Proteintech, Rosemont, IL, USA), rabbit polyclonal anti-TAFIa (1:100; 10672-1-AP, Proteintech), mouse monoclonal anti-C1q (1:100; ab71940, Abcam, Cambridge, UK), rat monoclonal anti-C3a (1:100; HM1072, Hycult Biotech, Wayne, PA, USA), rabbit polyclonal anti-C3b (1:100; GTX101316, GeneTex), rabbit polyclonal anti-C5a (1:100; bs-0324R, Bioss Antibodies, Woburn, MA, USA), mouse monoclonal anti-C5b (1:100; ab66768, Abcam), rabbit monoclonal anti-Wnt3a (1:100; #2721, Cell Signaling Technology, Danvers, MA, USA), rabbit polyclonal anti-Wnt5a (1:100; ab235966, Abcam), rabbit polyclonal anti-LRP5 (1:100; 24899-1-AP, Proteintech), rabbit polyclonal anti-Frizzled-5 (1:100; 21519-1-AP, Proteintech), goat polyclonal anti-TM (1:100; AF3894, R&D systems, Minneapolis, MN, USA), mouse monoclonal anti-Robo4 (1:50; sc-166872, Santa Cruz Biotechnology, Santa Cruz, CA, USA), rabbit monoclonal anti-claudin 5 (1:100; ab131259, Abcam), and rabbit monoclonal anti-E-cadherin (1:100; #3195, Cell Signaling Technology).

Sections were then incubated with the appropriate secondary antibodies (1:30 dilution; fluorescein isothiocyanate-conjugated anti-rabbit, anti-mouse, anti-rat, or anti-goat secondary antibody; Dako Cytomation, Glostrup, Denmark) for 2 h in the dark. Fluorescence intensity was quantified from five random fields of constant area using ImageJ software (version 2.1.53; NIH, Bethesda, MD, USA). The original files were converted to monochrome 8-bit images, and an arbitrary fluorescence intensity threshold was set. Areas exceeding the threshold (referred to as “intensity”) were measured for each sample.

### 4.5. Measurement of the Levels of Tumor-Associated Factors Using ELISA

Blood, liver, and aortic samples were collected at the end of the experiment. Plasma was separated from blood samples by centrifugation at 3000× *g* at 4 °C for 10 min., and the supernatant was used for further analysis. Plasma levels of uPA, IL-6, MMP-2, and MMP-9 were determined using commercially available ELISA kits according to the manufacturer’s instructions: uPA (IMSUPAKTT, Innovative Research Inc., Novi, MI, USA), IL-6 (M6000B; R&D Systems), MMP-2 (ab254516; Abcam), and MMP-9 (ab253227; Abcam).

Liver and aortic samples were homogenized at 15,000× *g* for 15 min at 4 °C (Tomy MX-201; Tomy Digital Biology, Tokyo, Japan), and the supernatant was collected for analysis. The levels of PAR1, PAR2, TF, TNF-α, TGF-β1, Ang-2, bFGF, β-catenin, vimentin, fibronectin, and Snail-1 in tissues were determined using commercially available ELISA kits: PAR1 (MBS753326, MyBioSource, San Diego, CA, USA), PAR2 (MBS4501658; MyBioSource), TF (ab214091; Abcam), TNF-α (KE10002, Proteintech), TGFβ1 (E-EL-M0051, Elabscience, Houston, TX, USA), Ang-2 (MANG20, R&D Systems), bFGF (bs-0217R, Bioss Antibodies), β-catenin (ADI-900-135; Enzo Life Sciences, Executive Blvd Farmingdale, NY, USA), fibronectin (OKCD05702, Aviva Systems Biology, San Diego, CA, USA), vimentin (ELK3731, ELK Biotechnology, Denver, CO, USA), and Snail-1 (LS-F2317-1, LS Bio, Shirley, MA, USA). The optical density was measured using a microplate reader (Molecular Devices, Sunnyvale, CA, USA).

### 4.6. Western Blotting Analysis of the Lungs and Aorta

Lung and aortic samples were homogenized in lysis buffer (Kurabo Industries, Osaka, Japan) and centrifuged to obtain the supernatants. Western blotting was performed as previously described [38]. After electrophoresis, membranes were incubated with primary antibodies against Ly6G (neutrophil activation marker, 1:1000; 551459, BD Biosciences, Franklin Lakes, NJ, USA), citH3 (neutrophil activation marker, 1:1000; ab281584, Abcam), PAD4 (neutrophil activation marker, 1:1000; ab214810, Abcam), and β-actin (1:5000; #58169, Cell Signaling) for 1 h at room temperature. β-actin was used as a loading control.

Membranes were washed and incubated with horseradish peroxidase-conjugated secondary antibodies (Novex, Frederick, MD, USA). Immune complexes were detected using ImmunoStar Zeta reagent (Wako Pure Chemical Industries, Osaka, Japan), and images were acquired using Multi Gauge software v3.0 (Fujifilm, Greenwood, SC, USA).

### 4.7. Statistical Analysis

All data are presented as mean ± standard deviation (SD). Microsoft Excel 2010 (Microsoft Corp., Redmond, WA, USA) and SPSS version 20 (SPSS Inc., Chicago, IL, USA) were used for statistical analysis. One-way analysis of variance followed by Tukey’s post hoc test was performed. Results were considered statistically significant at α = 0.05 (*p* < 0.05) or α = 0.01 (*p* < 0.01).

### 4.8. Ethical Statement for Animal Studies

All animal experiments were conducted in strict accordance with the recommendations of the Suzuka University of Medical Science Animal Experiment Ethics Committee (Approval no. 84) and prepared according to the guidelines of the Ministry of Education, Culture, Sports, Science, and Technology of Japan. Surgery was performed under pentobarbital anesthesia, and every effort was made to minimize pain.

## Figures and Tables

**Table 1:** Effect of RS ingestion on plasma and lung factors related to invasion and metastasis in mice implanted with B16 cells.

Factors in Plasma and Lungs	Control Group	RS Group	M + W Group	M + RS Group
IL-6 (pg/mL plasma)	19.0 ± 6.4	20.7 ± 7.2	136.9 ± 36.4 **	44.6 ± 12.7 ^##^
uPA (ng/mL plasma)	5.6 ± 1.5	7.2 ± 2.1	25.7 ± 5.5 **	15.6 ± 1.9 *^#^
MMP-2 (pg/mL plasma)	58.2 ± 18.7	61.1 ± 19.5	398.9 ± 61.5 **	137.5 ± 48.3 ^##^
MMP-9 (pg/mL plasma)	50.1 ± 18.0	36.1 ± 10.6	173.6 ± 59.5 **	83.4 ± 18.6 ^#^
PAR1 (ng/mg lung)	16.6 ± 6.5	15.2 ± 5.2	79.2 ± 9.9 **	53.2 ± 6.1 ^##^
PAR2 (ng/mg lung)	22.0 ± 6.5	17.1 ± 3.4	104.3 ± 13.5 **	93.3 ± 11.5
TGFβ1 (pg/mg lung)	7.5 ± 1.8	7.6 ± 1.4	20.3 ± 3.4 **	17.6 ± 3.9

Abbreviations: RS, rhamnan sulfate; M + W, melanocytes + water; M + RS, melanocytes + RS; IL-6, interleukin-6; uPA, urokinase-type plasminogen activator; MMP, matrix metalloprotease; PAR, protease-activated receptor; TGFβ1, transforming growth factor-β1. Each value represents the mean ± SD. Values shown as * *p* < 0.05 and ** *p* < 0.01 indicate significant differences against control group by Tukey’s test. Values shown as ^#^ *p* < 0.05 and ^##^ *p* < 0.01 indicate significant differences compared to the M + W group by Tukey’s test.

**Table 2:** Effect of RS ingestion on the levels of inflammation-, coagulation-, and fibrinolysis-related factors in lungs and aorta of mice implanted with B16 melanoma cells.

Factors in Lungs	Control Group	RS Group	M + W Group	M + RS Group
TNFα (pg/100 mg lung)	8.6 ± 3.0	7.0 ± 2.0	53.7 ± 9.6 **	33.2 ± 5.5 **^##^
TF (pg/100 mg lung)	17.7 ± 6.7	13.2 ± 3.9	72.6 ± 12.5 **	36.2 ± 5.2 *^##^
Thrombin (intensity)	9.1 ± 1.7	17.5 ± 3.6	14.3 ± 2.6	44.6 ± 5.6 **^##^
TM (intensity)	12.5 ± 2.3	16.2 ± 3.8	15.2 ± 2.6	49.9 ± 3.6 **^##^
Thrombin-TM (intensity)	6.7 ± 2.5	6.2 ± 1.0	9.6 ± 1.5	30.3 ± 5.4 **^##^
Plasmin (intensity)	12.6 ± 3.9	25.9 ± 5.4 *	9.9 ± 1.1	46.6 ± 7.8 **^##^
TAFl (intensity)	5.4 ± 2.0	5.9 ± 1.8	19.6 ± 2.9 **	3.4 ± 0.6 ^##^
TAFla (intensity)	3.2 ± 0.7	3.3 ± 0.6	1.9 ± 0.3	32.9 ± 4.1 **^##^
**Factors in Aorta**				
TNFα (pg/100 mg aorta)	5.2 ± 2.8	4.5 ± 1.0	40.5 ± 8.8 **	21.4 ± 6.9 ^##^
TF (pg/100 mg aorta)	18.0 ± 6.0	17.1 ± 4.5	84.9 ± 12.1 **	41.3 ± 8.0 ^##^
Thrombin (intensity)	5.7 ± 2.1	10.8 ± 1.3	4.2 ± 1.2	38.8 ± 4.8 **^##^
TM (intensity)	6.5 ± 0.9	11.5 ± 3.0	3.4 ± 0.9	45.4 ± 5.1 **^##^
Thrombin-TM (intensity)	3.0 ± 0.5	10.2 ± 1.6	4.0 ± 2.3	28.1 ± 7.0 **^##^
Plasmin (intensity)	2.9 ± 1.1	8.2 ± 1.2	1.2 ± 0.8	45.8 ± 5.5 **^##^
TAFl (intensity)	25.6 ± 5.1	28.2 ± 4.2	54.7 ± 9.1 **	9.7 ± 1.9 *^##^
TAFla (intensity)	2.3 ± 0.6	2.8 ± 0.6	1.7 ± 0.4	24.6 ± 7.7 **

Abbreviations: RS, rhamnan sulfate; M + W, melanocytes + water; M + RS, melanocytes + RS; TNFα, tumor necrosis factor α; TF, tissue factor; TM, thrombomodulin; TAFI, thrombin-activatable fibrinolysis inhibitor; TAFIa, activated TAFI. Each value represents the mean ± SD. Values shown as * *p* < 0.05 and ** *p* < 0.01 indicate significant differences compared to the control group by Tukey’s test. Values shown as ^##^ *p* < 0.01 indicate significant differences against the M + W group by Tukey’s test.

**Table 3:** Effect of RS ingestion on the levels of leukocyte-related factors in lungs and aorta of mice implanted with B16 melanoma cells.

Factors in Lungs	Control Group	RS Group	M + W Group	M + RS Group
F4/80 (intensity)	5.7 ± 2.4	5.7 ± 2.6	28.3 ± 7.1 **	18.1 ± 2.9 **^#^
Ly6G/β-actin (ratio)	1.0 ± 0.3	1.1 ± 0.2	2.4 ± 0.6 **	1.2 ± 0.3 ^##^
PAD4/β-actin (ratio)	0.5 ± 0.2	0.4 ± 0.2	0.7 ± 0.2 **	0.3 ± 0.1 ^##^
cisH3/β-actin (ratio)	0.2 ± 0.1	0.2 ± 0.1	0.5 ± 0.2 **	0.4 ± 0.1 ^#^
**Factors in Aorta**				
F4/80 (intensity)	3.0 ± 1.1	3.3 ± 1.1	56.5 ± 6.7 **	41.7 ± 5.8 **^#^
Ly6G/β-actin (ratio)	0.8 ± 0.2	0.8 ± 0.2	1.3 ± 0.2 **	1.1 ± 0.2
PAD4/β-actin (ratio)	0.2 ± 0.1	0.2 ± 0.1	0.4 ± 0.1 **	0.2 ± 0.1 ^##^
cisH3/β-actin (ratio)	0.2 ± 0.1	0.2 ± 0.1	0.8 ± 0.2 **	0.4 ± 0.1 *^##^

Abbreviations: RS, rhamnan sulfate; M + W, melanocytes + water; M + RS, melanocytes + RS; Ly6G, lymphocyte antigen 6 complex locus G; PAD4, protein-arginine deiminase type-4; cisH3, cytokine-inducible SH2-containing protein-3. Each value represents the mean ± SD. Values shown as * *p* < 0.05, ** *p* < 0.01 indicate significant differences compared to the control group by Tukey’s test. Values shown as ^#^ *p* < 0.05 and ^##^ *p* < 0.01 indicate significant differences compared to the M + W group by Tukey’s test.

**Table 4:** Effect of RS ingestion on the complement factors in lungs and aorta of mice implanted with B16 melanoma cells.

Factors in Lungs	Control Group	RS Group	M + W Group	M + RS Group
C1q (intensity)	12.5 ± 4.8	12.6 ± 3.6	39.7 ± 7.1 **	33.9 ± 4.6 **^#^
C3a (intensity)	23.9 ± 4.6	22.9 ± 5.4	43.8 ± 5.0 **	25.2 ± 7.5 ^##^
C3b (intensity)	21.8 ± 5.5	26.7 ± 5.5	43.1 ± 7.4 **	40.3 ± 6.8 **
C5a (intensity)	3.3 ± 0.9	3.2 ± 1.2	28.3 ± 9.7 **	14.8 ± 3.5 **^#^
C5b (intensity)	2.5 ± 1.0	2.0 ± 0.4	24.0 ± 6.1 **	21.6 ± 3.6 **
**Factors in Aorta**				
C1q (intensity)	12.9 ± 3.7	16.4 ± 3.0	50.1 ± 4.0 **	42.4 ± 5.3 **^#^
C3a (intensity)	3.1 ± 1.2	2.7 ± 0.9	36.0 ± 5.1 **	7.1 ± 2.3 ^##^
C3b (intensity)	6.5 ± 3.8	6.8 ± 1.7	51.2 ± 7.9 **	47.8 ± 4.2 **
C5a (intensity)	1.7 ± 0.7	1.8 ± 0.8	25.2 ± 6.6 **	4.1 ± 1.2 ^##^
C5b (intensity)	1.3 ± 0.4	1.3 ± 0.5	29.8 ± 5.6 **	25.0 ± 6.7 **

Abbreviations: RS, rhamnan sulfate; M + W, melanocytes + water; M + RS, melanocytes + RS; C1q, complement component 1q; C3a, complement component 3a; C3b, complement component 3b; C5a, complement component 5a; C5b, complement component 5b. Each value represents the mean ± SD. Values shown as ** *p* < 0.01 indicate significant differences compared to the control group by Tukey’s test. Values shown as ^#^ *p* < 0.05 and ^##^ *p* < 0.01 indicate significant differences compared to the M + W group by Tukey’s test.

**Table 5:** Effect of RS ingestion on the levels of tumor angiogenesis and invasion-related factors in lungs and aorta of mice implanted with B16 melanoma cells.

Factors in Lungs	Control Group	RS Group	M + W Group	M + RS Group
Ang-2 (ng/100 mg lung)	8.4 ± 3.4	6.0 ± 2.3	47.2 ± 9.4 **	18.1 ± 7.7 ^##^
bFGF (pg/100 mg lung)	5.4 ± 1.7	5.4 ± 1.8	65.9 ± 18.3 **	38.8 ± 8.3 ^#^
Robo4 (intensity)	4.8 ± 1.0	5.0 ± 1.6	24.3 ± 4.9 **	17.8 ± 4.1 **^#^
Claudin 5 (intensity)	17.7 ± 4.1	20.3 ± 3.8	4.1 ± 1.5 **	10.3 ± 1.3 ^##^
E-cadherin (intensity)	17.7 ± 3.8	18.1 ± 2.3	3.3 ± 1.0 **	18.5 ± 3.1 ^##^
**Factors in Aorta**				
Ang-2 (ng/100 mg aorta)	4.4 ± 1.6	3.2 ± 0.5	21.8 ± 5.2 **	9.8 ± 1.9 *^##^
bFGF (pg/100 mg aorta)	4.8 ± 1.6	4.2 ± 1.2	76.3 ± 16.2 **	18.5 ± 7.8 **^##^
Robo4 (intensity)	2.3 ± 0.7	3.0 ± 0.8	30.0 ± 5.4 **	12.8 ± 3.3 *^##^
Claudin 5 (intensity)	15.6 ± 3.0	13.0 ± 2.4	1.9 ± 0.5 **	15.9 ± 2.9 ^##^
E-cadherin (intensity)	12.0 ± 2.1	12.5 ± 2.8	1.7 ± 0.4 **	13.3 ± 1.9 ^##^

Abbreviations: RS, rhamnan sulfate; M + W, melanocytes + water; M + RS, melanocytes + RS; Ang-2, angiopoietin 2; bFGF, basic fibroblast growth factor; Robo4, Roundabout homolog 4; E-cadherin, epithelial cadherin. Each value represents the mean ± SD. Values shown as * *p* < 0.05, ** *p* < 0.01 indicate significant differences compared to the control group by Tukey’s test. Values shown as ^#^ *p* < 0.05 and ^##^ *p* < 0.01 indicate significant differences compared to the M + W group by Tukey’s test.

**Table 6:** Effect of RS ingestion on the EMT-related factors in lungs and aorta of mice implanted with B16 melanoma cells.

Factors in Lungs	Control Group	RS Group	M + W Group	M + RS Group
β-catenin (ng/100 mg lung)	0.7 ± 0.1	0.6 ± 0.0	1.2 ± 0.3 *	0.9 ± 0.2 ^#^
Fibronectin (ng/100 mg lung)	19.8 ± 6.7	16.6 ± 6.9	54.2 ± 13.0 **	38.4 ± 7.8 ^#^
Snail-1 (ng/100 mg lung)	9.4 ± 3.1	8.0 ± 2.2	32.1 ± 7.2 **	13.5 ± 2.6 ^##^
Vimentin (ng/100 mg lung)	12.5 ± 4.5	10.6 ± 2.8	61.6 ± 18.9 **	40.6 ± 7.3 ^#^
Wnt3a (intensity)	9.8 ± 1.7	10.0 ± 1.5	62.4 ± 7.2 **	11.2 ± 1.5 ^##^
Wnt5a (intensity)	9.3 ± 1.7	8.0 ± 1.9	8.1 ± 1.1	42.2 ± 6.0 ^##^
LRP5 (intensity)	3.2 ± 0.9	3.8 ± 2.0	22.1 ± 5.0 **	9.6 ± 1.6 **^##^
Frizzled-5 (intensity)	12.8 ± 2.6	9.6 ± 1.5	20.6 ± 2.8 **	14.8 ± 2.9 ^#^
**Factors in Aorta**				
β-catenin (ng/100 mg aorta)	0.3 ± 0.2	0.2 ± 0.1	0.7 ± 0.2 **	0.5 ± 0.1 ^#^
fibronectin (ng/100 mg aorta)	14.4 ± 4.6	11.9 ± 3.9	37.4 ± 6.1 **	21.1 ± 9.3 ^##^
Snail-1 (ng/100 mg aorta)	7.2 ± 1.4	6.2 ± 1.1	27.1 ± 6.4 **	13.4 ± 5.9 *^##^
Vimentin (ng/100 mg aorta)	6.9 ± 2.1	5.6 ± 1.6	48.2 ± 11.4 **	34.5 ± 5.3 **^#^
Wnt3a (intensity)	6.7 ± 2.8	8.0 ± 1.5	42.3 ± 10.3 **	9.2 ± 1.3 ^##^
Wnt5a (intensity)	9.3 ± 1.7	10.0 ± 0.9	5.8 ± 1.2	64.5 ± 6.9 **^##^
LRP5 (intensity)	3.4 ± 1.2	3.5 ± 1.1	21.1 ± 5.3 **	6.6 ± 1.5 ^##^
Frizzled-5 (intensity)	5.7 ± 1.7	3.7 ± 1.6	20.4 ± 5.0 **	7.4 ± 2.0 ^##^

Abbreviations: RS, rhamnan sulfate; M + W, melanocytes + water; M + RS, melanocytes + RS; Snail-1, snail family transcriptional repressor 1; Wnt3a, wingless-type MMTV integration site family member 3A; Wnt5a, Wnt family member 5A; LRP5, low-density lipoprotein receptor-related protein 5. Each value represents the mean ± SD. Values shown as * *p* < 0.05 and ** *p* < 0.01 indicate significant differences compared to the control by Tukey’s test. Values shown as ^#^ *p* < 0.05 and ^##^ *p* < 0.01 indicate significant differences compared to the M + W group by Tukey’s test.

## Data Availability

The data presented in this study are available on request from the corresponding author, K.S., and author K.H.

## Abbreviations

The following abbreviations are used in this manuscript.

Ang-2	Angiopoietin-2
bFGF	Basic fibroblast growth factor
C1q	Complement component 1q
C3a	Complement component 3a
C3b	Complement component 3b
C5a	Complement component 5a
C5b	Complement component 5b
cisH3	Cytokine-inducible SH2-containing protein-3
DOAC	Direct oral anticoagulant
DOPA	3,4-dihydroxyphenylalanine
E-cadherin	Epithelial cadherin
ELISA	Enzyme-linked immunosorbent assay
EMT	Epithelial–mesenchymal transition
IL-6	Interleukin-6
LPS	Lipopolysaccharide
LRP5	Low-density lipoprotein receptor-related protein 5
Ly6G	Lymphocyte antigen 6 complex locus G
MMP	Matrix metalloprotease
PAD4	Protein-arginine deiminase type-4
PAR	Protease-activated receptor
Robo4	Roundabout homolog 4
RS	Rhamnan sulfate
Snail-1	Snail family transcriptional repressor 1
TAFI	Thrombin-activatable fibrinolysis inhibitor
TAFIa	Activated TAFI
TF	Tissue factor
TGFβ	Transforming growth factor-β
TM	Thrombomodulin
TNFα	Tumor necrosis factor α
uPA	Urokinase-type plasminogen activator
Wnt3a	Wingless-type MMTV integration site family member 3A
Wnt5a	Wnt family member 5A
